# Supplementary material for: Tailoring the thermal and electrical transport properties of graphene films by grain size engineering
Source: Nat Commun. 2017 Feb 16;8:14486. doi: 10.1038/ncomms14486 (PMC5316893; doi:10.1038/ncomms14486)
Supplement: Supplementary Information — Supplementary Figures, Supplementary Notes and Supplementary References [file ncomms14486-s1.pdf]

Supplementary Note 1 | XPS analysis of Pt substrates. To understand the growth behavior of graphene in our SACVD process, we used X-ray photoelectron spectroscopy (XPS) to study the carbon content in Pt substrates obtained after different growth steps (Supplementary Figs. 1 and 2). It was found that carbon atoms dissolved in the Pt substrate during the formation of a monolayer-dominated graphene film during the first step (Supplementary Fig. 2) because of the medium carbon solubility of Pt (0.07 wt.%)[1], which is higher than that of Cu (0.008 wt.%) but lower than Ni (0.3 wt.%) at 1000 °C[2]. The amount of carbon dissolved in Pt substrate decreased during the third step with the formation of small graphene domains. Considering the fact that no extra carbon was supplied in this step, this result is strong evidence that hydrogen can trap the pre-dissolved carbon to the surface of Pt, leading to segregation growth of graphene.

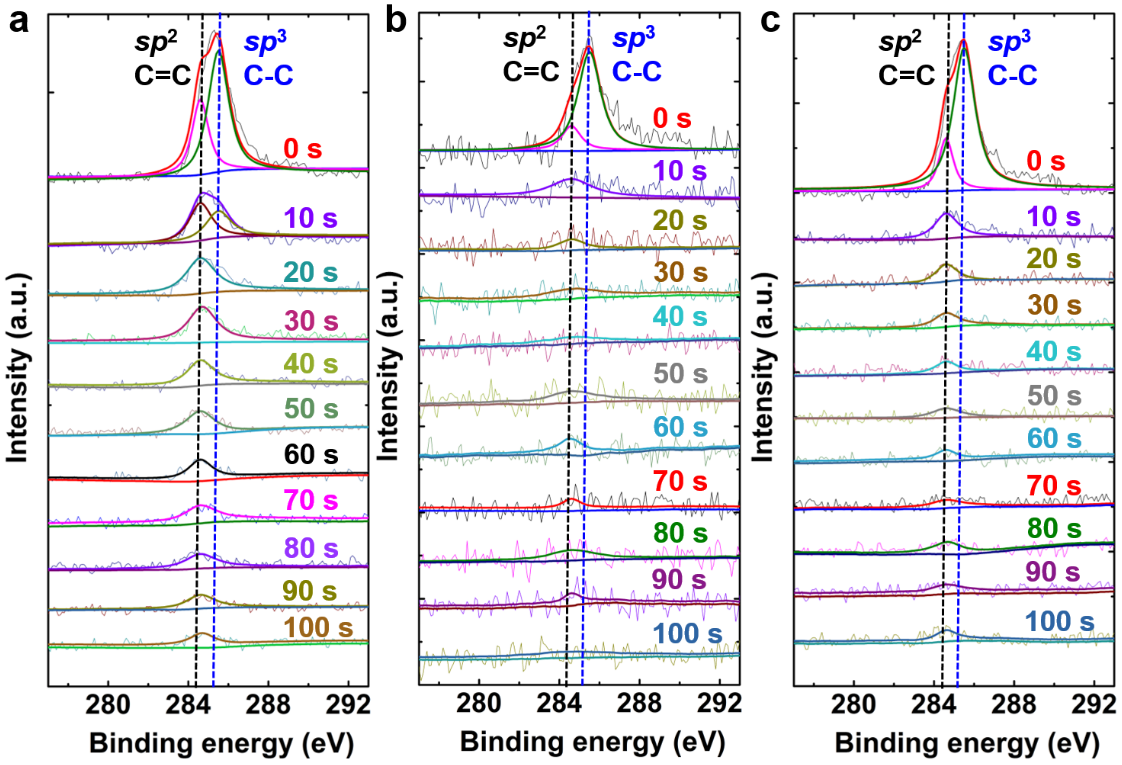

Supplementary Figure 1 | XPS C1*s* spectra taken from the surface to the interior of the Pt substrate after the different steps of film formation. To obtain the carbon content at different depths, 500 eV Ar ion sputtering was used to etch the Pt surface, with etching times from 0 s to

100 s. (**a**) Monolayer-dominated graphene films/Pt (obtained in step 1). (**b**) small graphene domains/Pt (obtained in step 3). (**c**) Strictly monolayer graphene films/Pt (obtained in step 4).

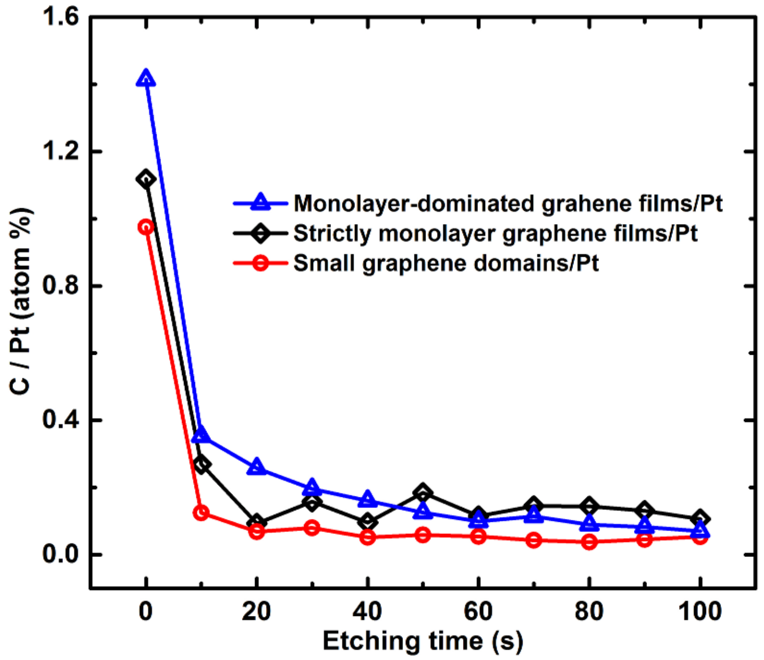

Supplementary Figure 2 | Carbon content in Pt substrates. Atomic ratio of carbon to platinum as a function of etching (500 eV Ar ion sputtering) time for monolayer-dominated graphene films/Pt obtained in step 1, blue line, small graphene domains/Pt obtained in step 3, red line and strictly monolayer graphene films/Pt obtained in step 4, black line.

Supplementary Note 2 | The influence of hydrogen flow rate and segregation temperature on CVD growth. We studied the influence of hydrogen on the growth behavior of graphene in the third step. If the flow rate of hydrogen was increased in this step, Supplementary Fig. 3, a continuous graphene film with irregular holes and rather isolated small domains were formed, confirming the trapping effect of hydrogen on the dissolved carbon mentioned above. The formation of holes is attributed to the etching of hydrogen[3-5]. However, decreasing the flow rate

of hydrogen led to the formation of multilayer graphene domains. Therefore, the trapping and etching effects of hydrogen are two competitive processes, and must be balanced during the segregation growth process to ensure the formation of isolated monolayer domains. The reaction temperature in the segregation step is the only factor that determines the domain density, which increases with decreasing reaction temperature (Fig. 2a-d in the main text and Supplementary Fig. 4). When we fixed the flow rate of hydrogen during this step, the small graphene domains obtained shrank with increasing reaction time because of the etching effect of hydrogen, which provides an efficient way to produce graphene quantum dots by CVD (Supplementary Fig. 5). For example, graphene quantum dots with a size smaller than 10 nm can be obtained by extending the reaction time (Supplementary Fig. 5d).

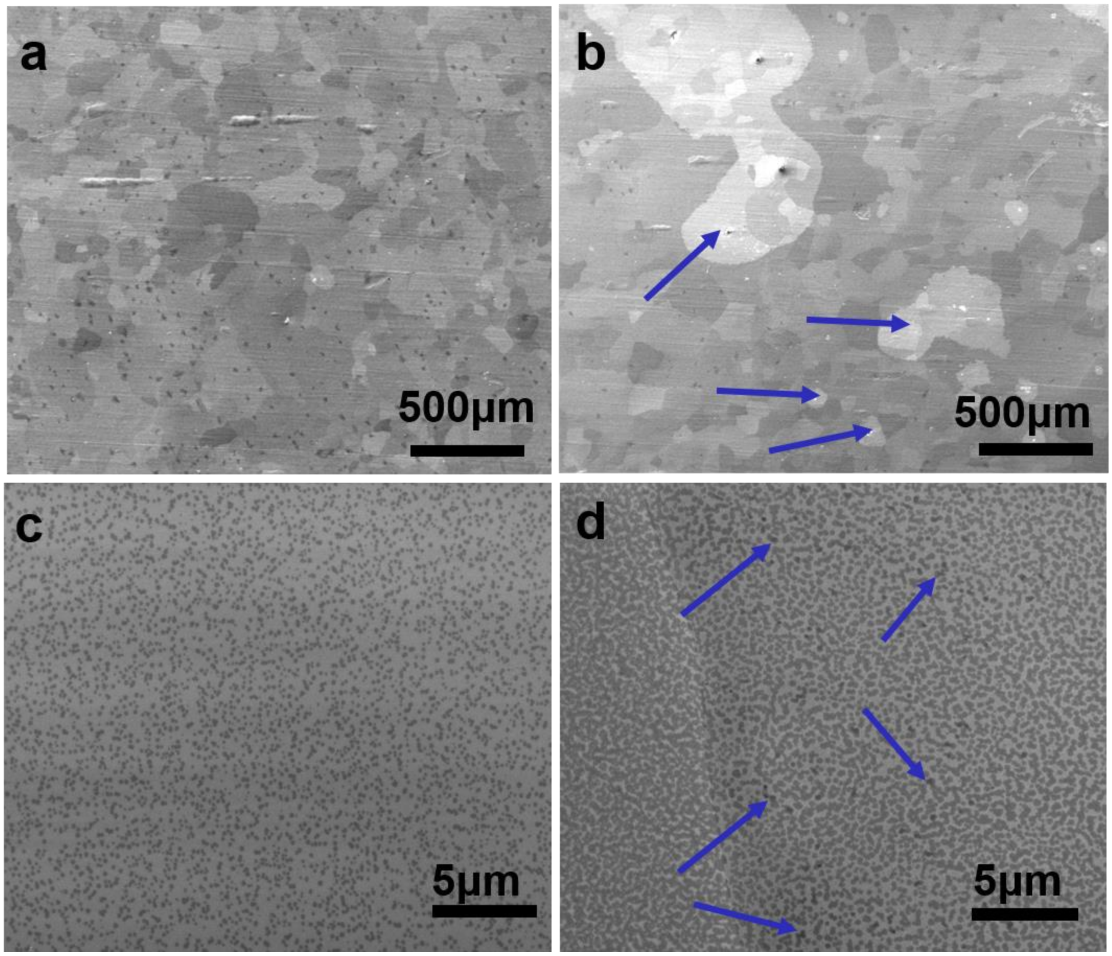

Supplementary Figure 3 | SEM images of graphene on a Pt substrate obtained at the different stages. (**a**) Graphene films obtained with a mixture of hydrogen (700 sccm) and methane (7 sccm) for 5 min at 1000 ºC in the first step. (**b**) Holey graphene films obtained from **a** after argon treatment (700 sccm) for 20 min in the second step, followed by segregation with a mixture of hydrogen (200 sccm) and argon (500 sccm) for 10 min in the third step. Some holes are indicated by blue arrows. (**c**) Uniform small monolayer graphene domains obtained after argon treatment (700 sccm) for 20 min in the second step from **a**, followed by segregation with a mixture of hydrogen (15 sccm) and argon (700 sccm) for 20 min in the third step. (**d**) Monolayer and few-layer graphene domains obtained from **a** after argon treatment (700 sccm) for 20 min in the second step, followed by segregation with a mixture of hydrogen (5 sccm) and argon (700 sccm) for 20 min in the third step. Some multilayers are indicated by blue arrows.

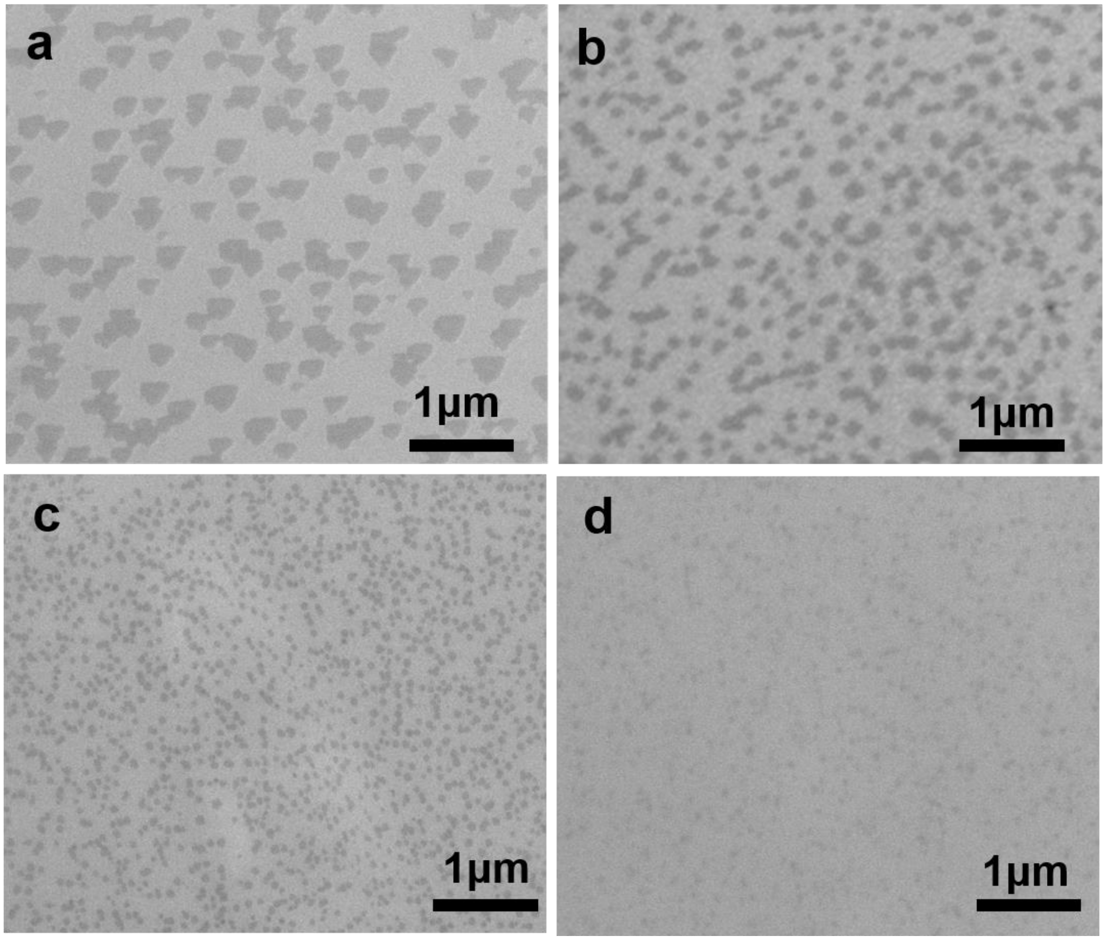

Supplementary Figure 4 | SEM images of isolated small graphene domains a Pt substrate obtained under different conditions.

(a) Step 1. Surface growth with 700 sccm hydrogen and 7 sccm methane for 3 min at 1040°C

Step 2. Thermal annealing in pure argon for 20 min at 1040°C

Step 3. Heated in a mixture of H_2 (20 sccm) and Ar (700 sccm) for 20 min at 1040 ºC

(b) Step 1. Surface growth with 700 sccm hydrogen and 7 sccm methane for 5 min at 1000°C

Step 2. Thermal annealing in pure argon for 20 min at 1000°C

Step 3. Heated in a mixture of H_2 (15 sccm) and Ar (700 sccm) for 20 min at 1000 ºC

(c) Step 1. Surface growth with 700 sccm hydrogen and 7 sccm methane for 8 min at 950°C

Step 2. Thermal annealing in pure argon for 20 min at 950°C

Step 3. Heated in a mixture of H_2 (10 sccm) and Ar (700 sccm) for 20 min at 950 ºC

(d) Step 1. Surface growth with 700 sccm hydrogen and 7 sccm methane for 10 min at 900°C

Step 2. Thermal annealing in pure argon for 20 min at 900°C

Step 3. Heated in a mixture of H_2 (5 sccm) and Ar (700 sccm) for 20 min at 900 ºC

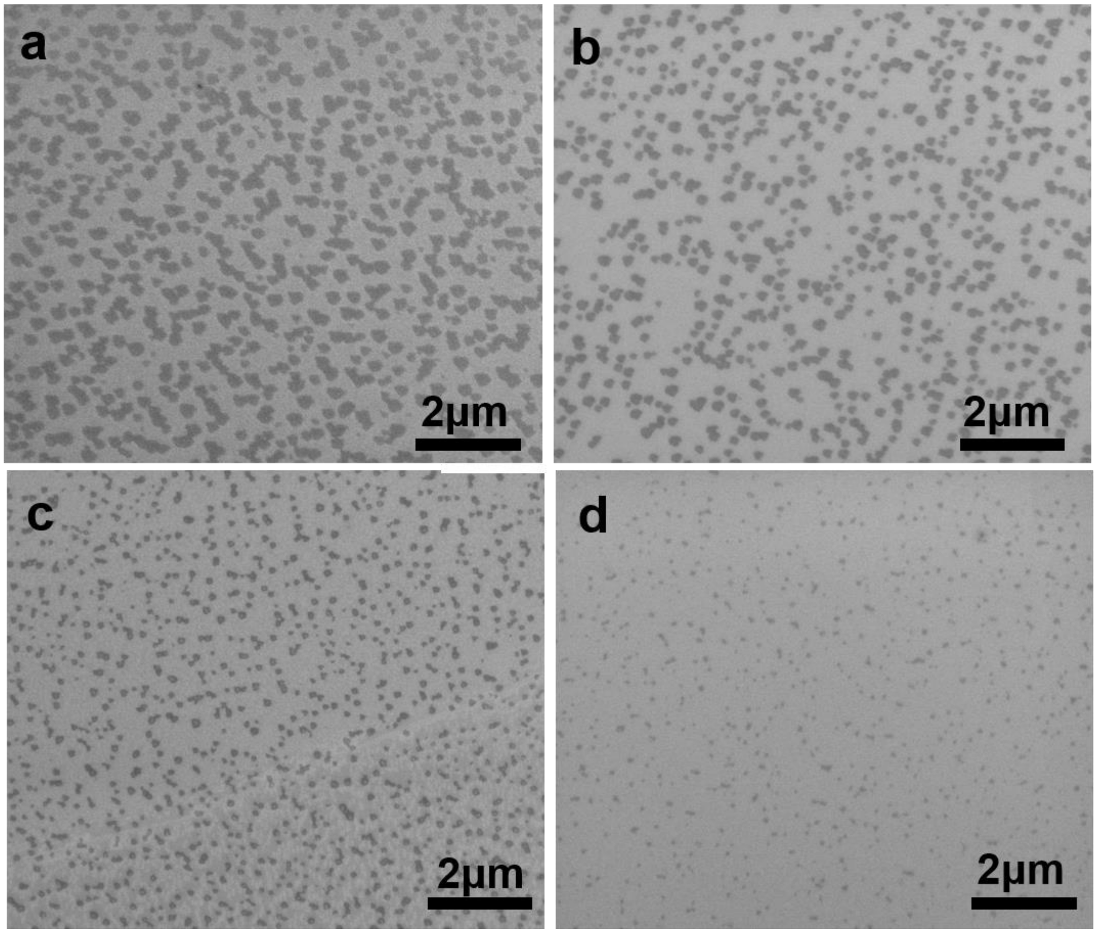

Supplementary Figure 5 | SEM images of the etched isolated small graphene domains. Graphene domains obtained after etching for 10 (**a**), 15 (**b**), 20 (**c**), and 30 (**d**) min in a mixture of hydrogen (15 sccm) and argon (700 sccm) at 1000 ºC. The original sample was prepared with

700 sccm hydrogen and 7 sccm methane for 5 min at 1000 ºC, followed by thermal annealing in pure argon for 20 min.

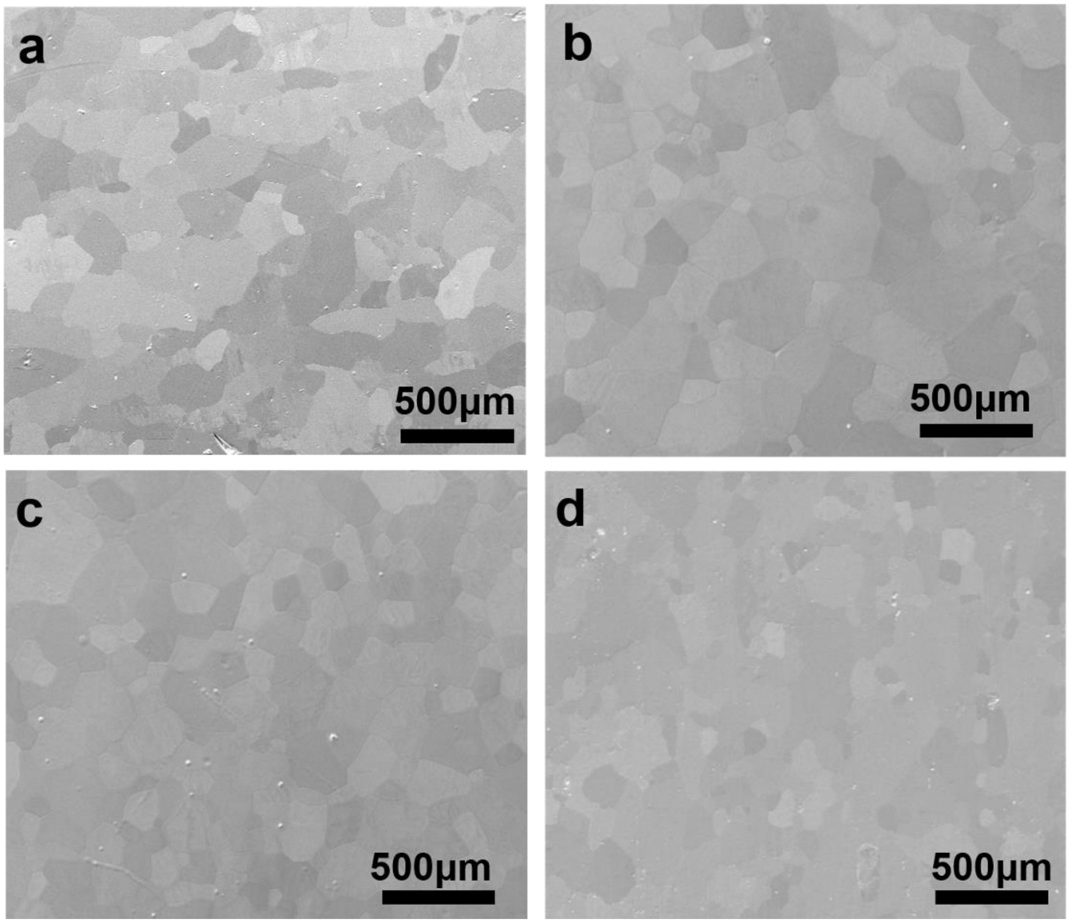

Supplementary Figure 6 | SEM images of uniform monolayer polycrystalline graphene films with different grain sizes. (**a**) ~200 nm, (**b**) ~500 nm, (**c**) ~700 nm, (**d**) ~1 μm.

Supplementary Note 3 | Surface growth of the small graphene domains. In the fourth step, the introduction a small flow of methane to change the graphene kinetic behavior from etching to growing, the isolated graphene domains expanded and eventually joined together to form

continuous graphene films with different grain sizes (Supplementary Fig. 6). It should be noted that the carbon content in this step is higher than that in the third step but still lower than that in the first step (Supplementary Fig. 2), ensuring the formation of strictly monolayer graphene films. Moreover, no additional layers were formed even by further extending the growth time, because the catalytic activity of Pt was suppressed after graphene coverage, indicating the self-limiting surface adsorption behavior of graphene in the fourth step.

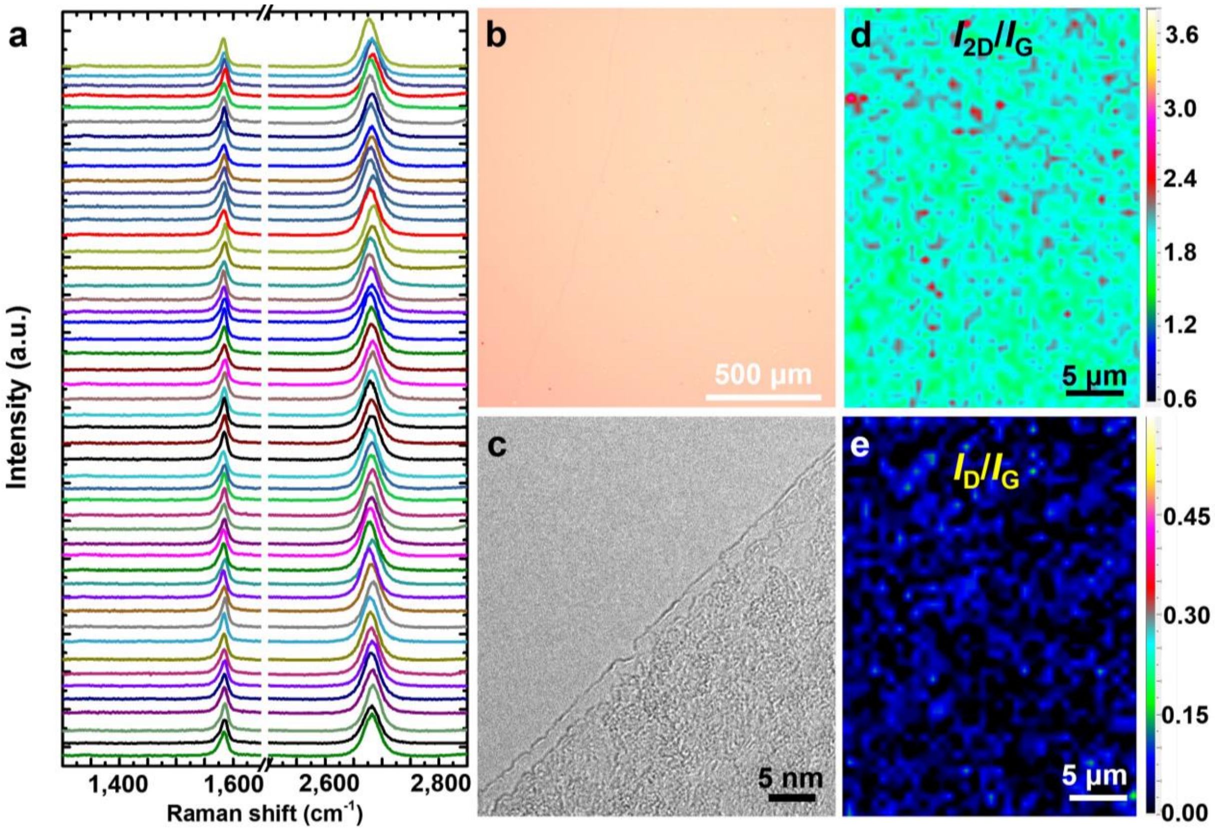

Supplementary Figure 7 | Structure characterization of the graphene films with ~200 nm-sized grains. (**a**) Raman spectra randomly acquired from more than 50 positions on a large-area graphene film. (**b**) Optical image of the graphene film transferred onto the SiO_2/Si

substrate. (**c**) HRTEM image of the edge of the graphene films. (**d**,**e**) Raman mappings of the intensity ratios of the 2D peak to the G peak (**d**) and the D peak to the G peak (**e**).

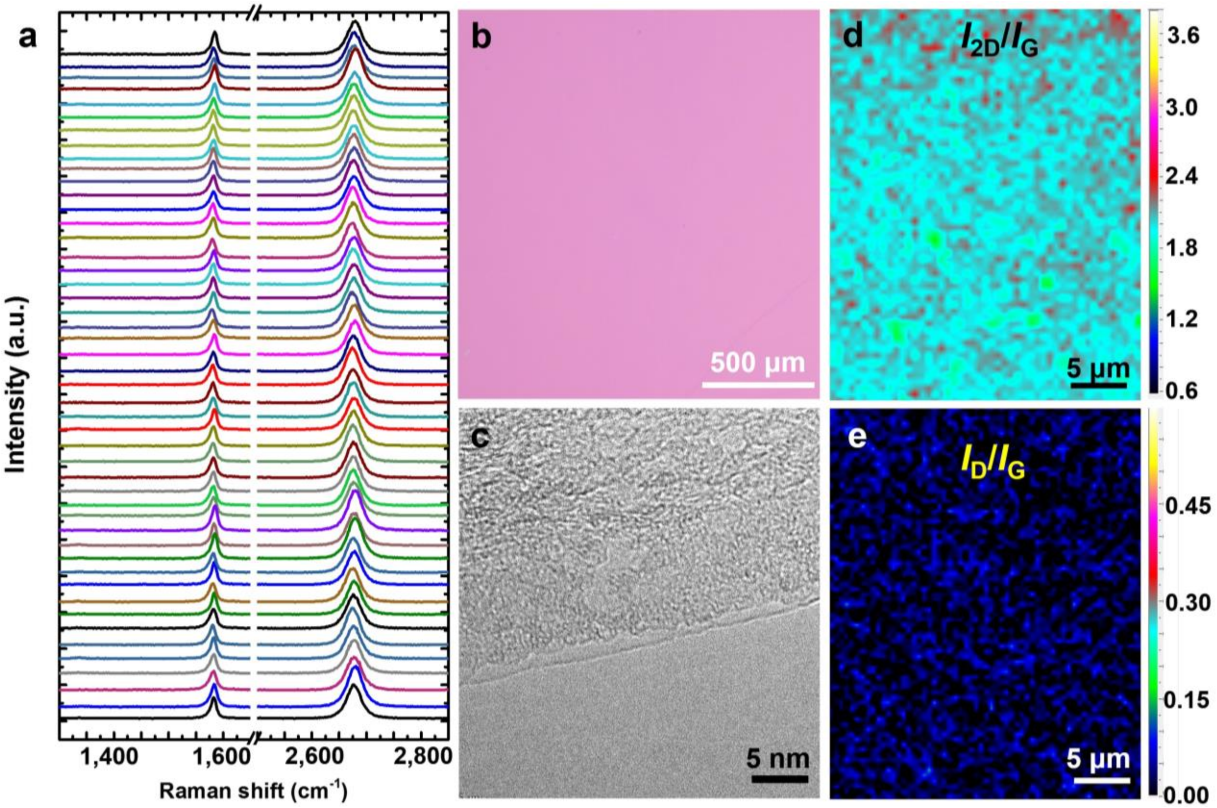

Supplementary Figure 8 | Structure characterization on the graphene films with ~1 μm-sized grains. (**a**) Raman spectra randomly acquired from more than 50 positions on a large-area graphene film. (**b**) Optical image of the graphene film transferred onto a SiO_2/Si substrate. (**c**) HRTEM image of the edge of the graphene film. (**d**,**e**) Raman mappings of the intensity ratios of the 2D peak to the G peak (**d**) and the D peak to the G peak (**e**).

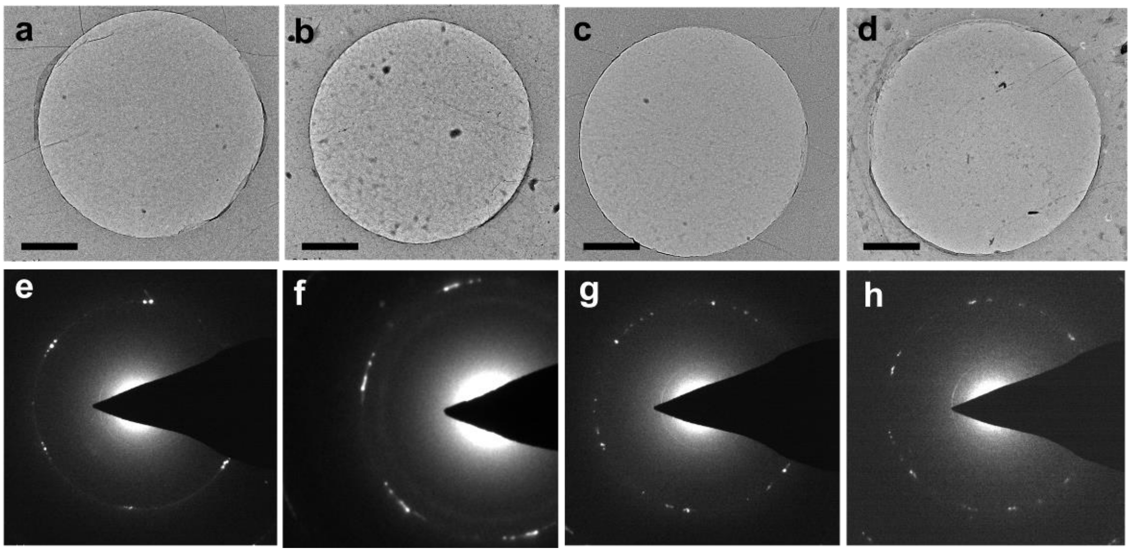

Supplementary Figure 9 | TEM images (**a**-**d**) and the corresponding selected-area electron diffraction patterns (**e**-**h**) of the polycrystalline graphene films shown in Fig. 2e-h in the main text. All the scale bars are 500 nm.

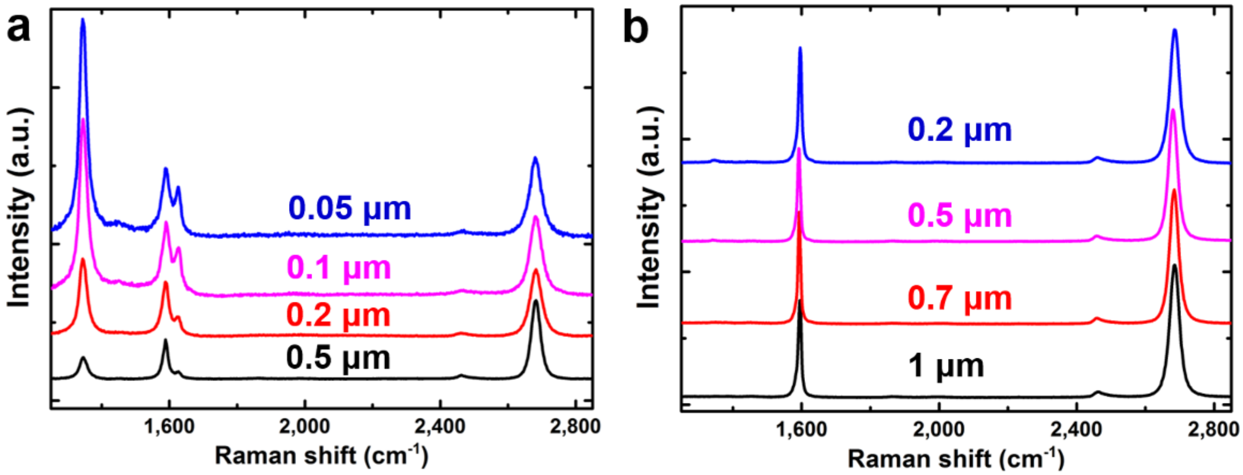

Supplementary Figure 10 | Raman spectra of (**a**) isolated small graphene domains formed in step 3 and (**b**) the corresponding polycrystalline graphene films formed in step 4, which were transferred onto SiO_2/Si substrates. The numbers above the lines show (**a**) the domain size and (**b**)

the grain size. All the isolated graphene domains show a very strong D peak whose intensity greatly increases with decreasing domain size, giving strong evidence that the D peaks originate from the domain edges. In sharp contrast, no visible D peaks are observed for all the graphene films, indicating that all the grains in the films are perfectly stitched together without gaps and no defects exist within the plane.

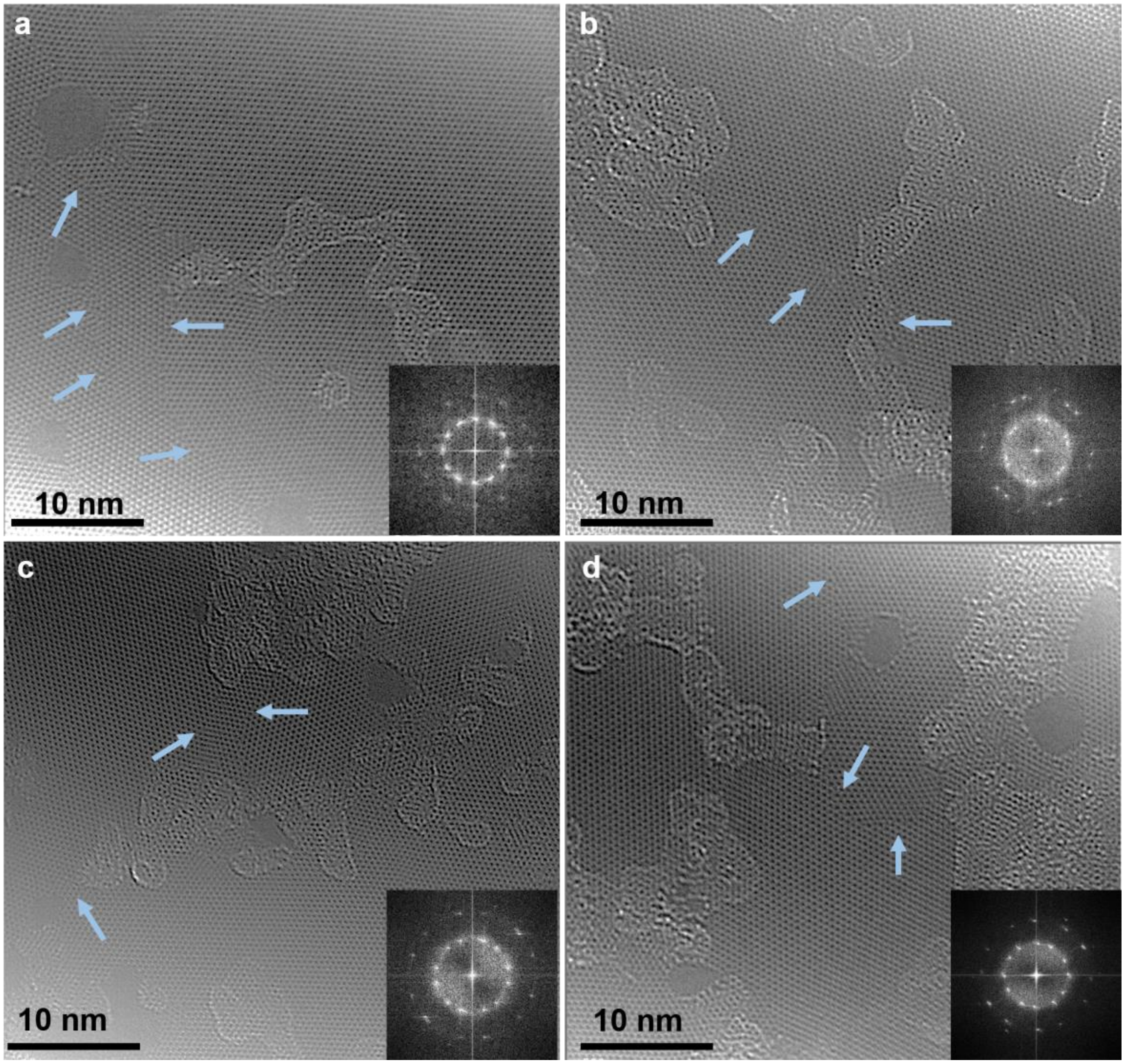

Supplementary Figure 11 | Large-area atomic resolution HRTEM images of the graphene films with grain size of ~200 nm (**a,b**) and ~700 nm (**c,d**). The insets are the corresponding fast

Fourier transformation patterns, showing polycrystalline structure. The grain boundaries are indicated by blue arrows. The contaminations on the surface of graphene films are PMMA residues, which are very common for the graphene transferred by PMMA. The holes were generated by long-time electron beam irradiation during HRTEM observations.

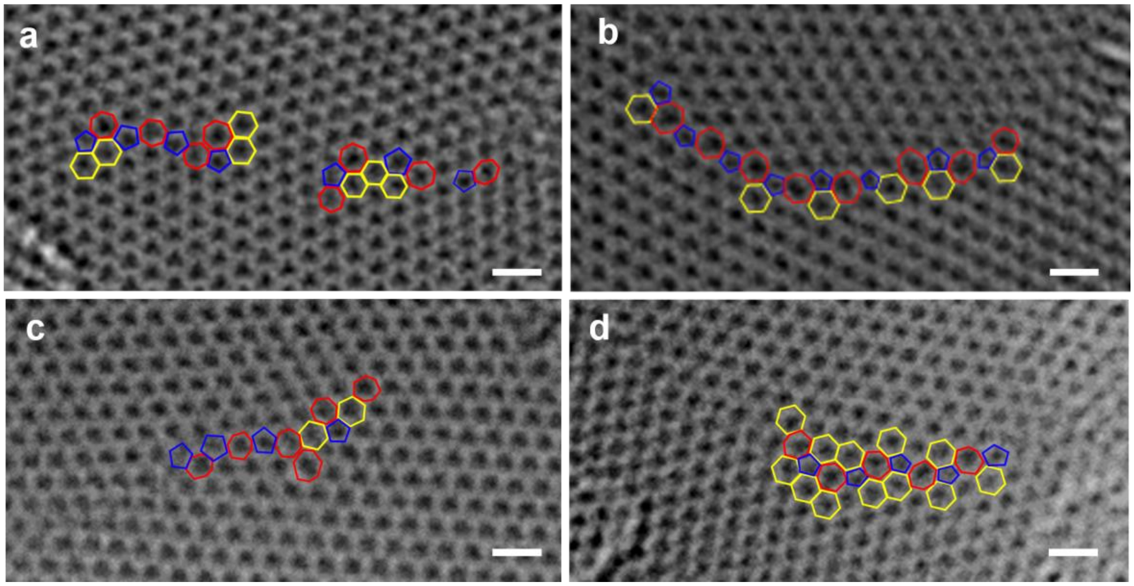

Supplementary Figure 12 | HRTEM images of the graphene films with grain size of ~200 nm (**a**,**c**) and ~700 nm (**b**,**d**). The pentagons (blue), heptagons (red) and hexagons (yellow) in the grain boundaries are outlined. All images were processed with an improved Wiener-Filtering to remove the noises. The scale bars are 1 nm.

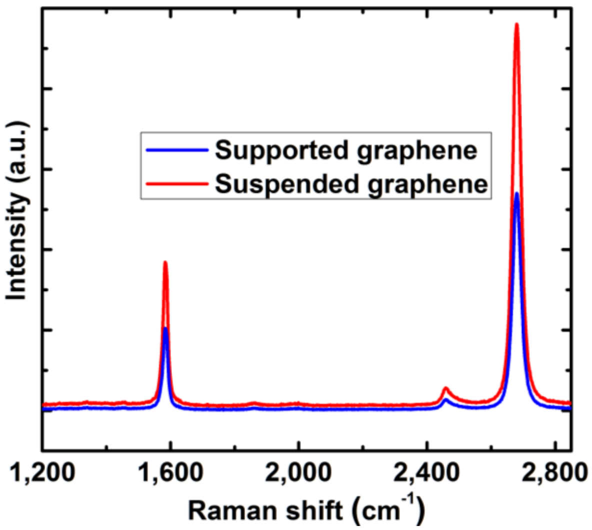

Supplementary Figure 13 | Typical Raman spectra of the supported and suspended graphene films. The intensity of the Raman spectrum obtained from the suspended area is much higher than that of the supported film and the D peak at 1340 cm^{-1} is almost invisible, indicating the intact and high-quality features of both supported and suspended graphene films.

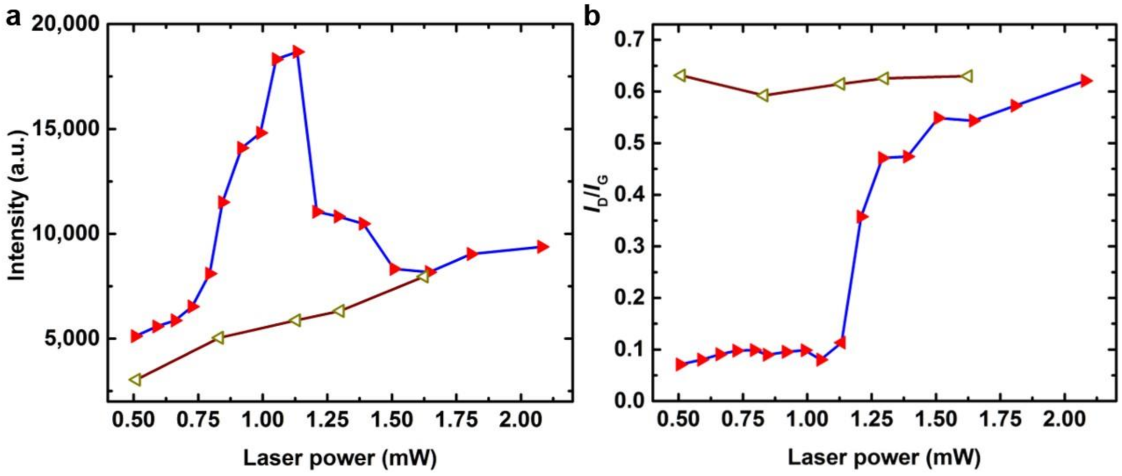

Supplementary Figure 14 | Thermal transport of graphene films with ~200 nm-sized grains. Intensity of the G peak (**a**) and I_D/I_G (**b**) as a function of laser power.

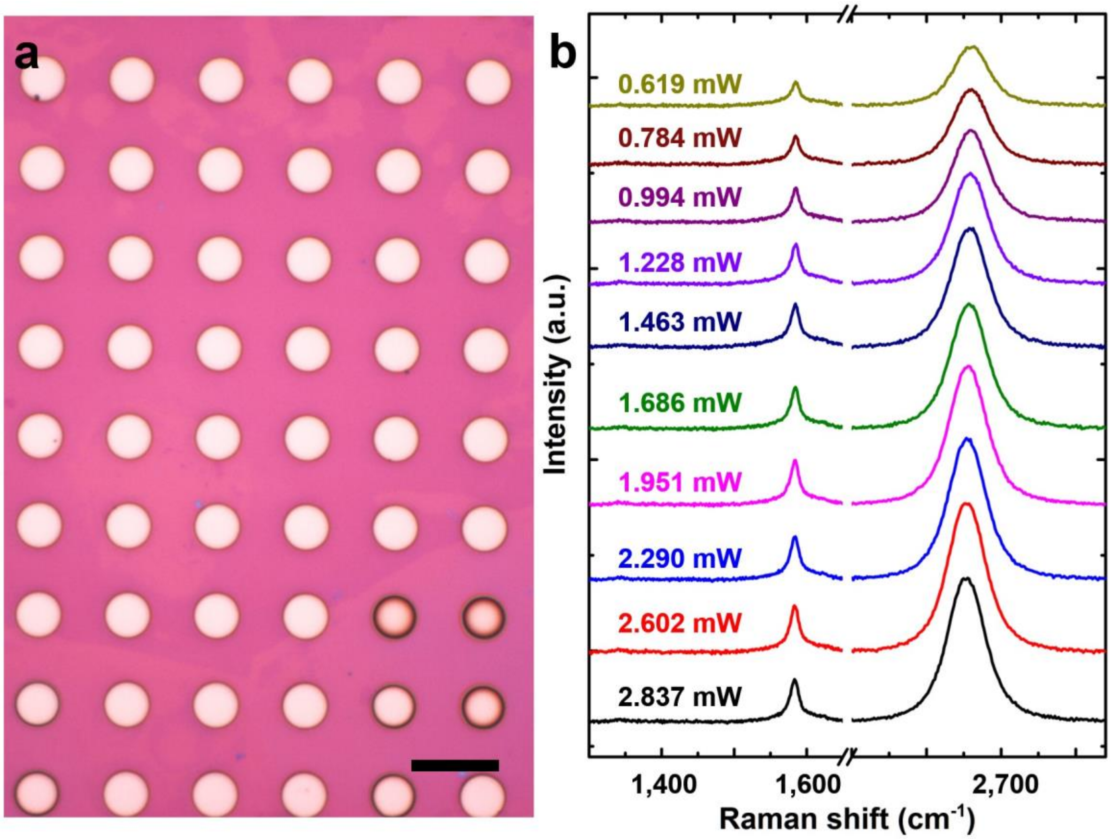

Supplementary Figure 15 | Optical image and Raman spectra of single-crystal graphene domain. (**a**) Optical image of single-crystal graphene domains transferred onto a holey SiO_2/Si substrate. The scale bar is 10 μm. (**b**) Raman spectra of a suspended single-crystal graphene domain excited with different power lasers. The absence of the D peak indicates that it remained intact during high power laser irradiation up to 2.8 mW.

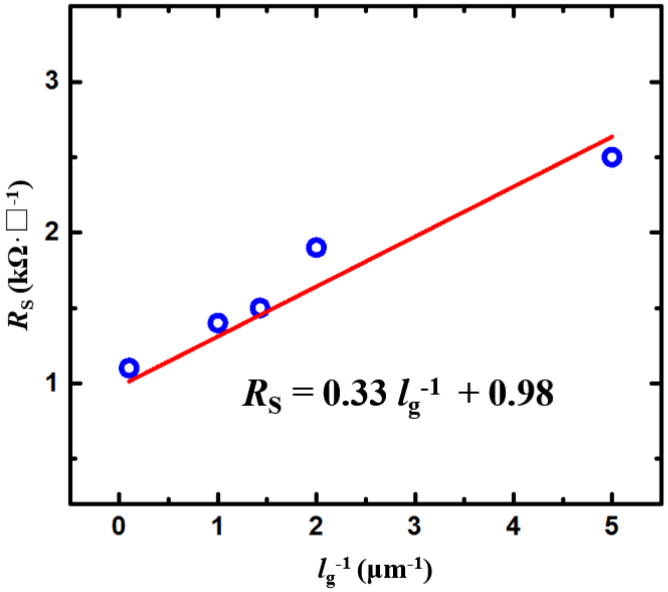

Supplementary Figure 16 | Electrical properties of graphene films with grain sizes ranging from ~200 nm to ~10 μm. Sheet resistance as a function of the inverse of grain size with a fit (red curve).

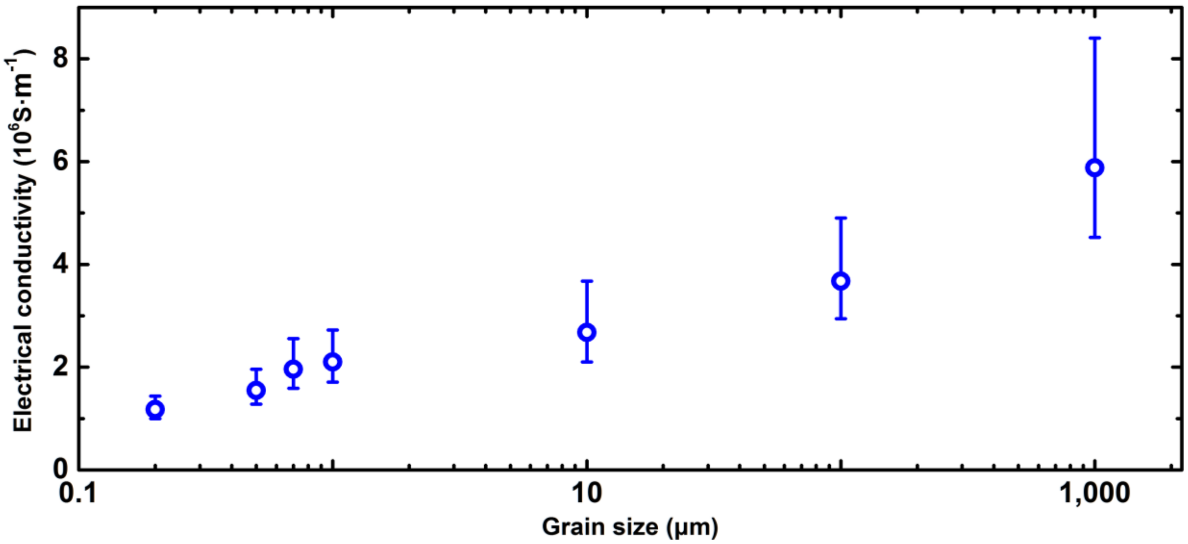

Supplementary Figure 17 | Electrical properties of graphene films with grain sizes ranging from ~200 nm to 1 mm. Electrical conductivity as a function of grain size, showing a weak

dependence. The error bars (s.e.m.) represent the electrical conductivity variation measured for the same sample. The electrical conductivity (σ) of the graphene films was calculated by $\sigma = R_s^{-1} h^{-1}$, where R_s is the sheet resistance of the polycrystalline films and h is the thickness of the graphene films.

Supplementary References

1. Siller, R. H., Oates, W. A. & McLellan, R. B. The solubility of carbon in palladium and platinum. *J. Less Common Met.* **16,** 71-73 (1968).
2. Li, X., Cai, W., Colombo, L. & Ruoff, R. S. Evolution of graphene growth on Ni and Cu by carbon isotope labeling. *Nano Lett.* **9,** 4268-4272 (2009).
3. Vlassiouk, I. *et al.* Role of hydrogen in chemical vapor deposition growth of large single-crystal graphene. *ACS Nano* **5,** 6069-6076 (2011).
4. Ma, T. *et al.* Edge-controlled growth and kinetics of single-crystal graphene domains by chemical vapor deposition. *Proc. Natl. Acad. Sci. U.S.A.* **110,** 20386-20391 (2013).
5. Ma, T. *et al.* Repeated growth-etching-regrowth for large-area defect-free single-crystal graphene by chemical vapor deposition. *ACS Nano* **8,** 12806-12813 (2014).
